# Juvenile Idiopathic Arthritis—The Rubik’s Cube of Pediatric Rheumatology

**DOI:** 10.3390/children12101319

**Published:** 2025-10-01

**Authors:** Olcay Y. Jones, Deborah K. McCurdy, Charles H. Spencer, Daniel J. Lovell

**Affiliations:** 1 Division of Pediatric Rheumatology, Department of Pediatrics, Walter Reed National Military Medical Center, Bethesda, MD 20889, USA; 2 Department of Pediatrics, Uniformed Services University of the Health Sciences, Bethesda, MD 20814, USA; 3 Division of Allergy, Immunology, and Rheumatology, Department of Pediatrics, University of California, Los Angeles, CA 90095, USA; 4 Pediatric Rheumatology, Madison, MS 53792, USA; 5 Division of Pediatric Rheumatology, Cincinnati Children’s Hospital Medical Center, Cincinnati, OH 45229, USA

**Keywords:** pediatric rheumatology, immunology, JIA

## Abstract

**Background/Objectives**: Juvenile Idiopathic Arthritis (JIA) is the most common autoimmune rheumatic disease in children and can vary in presentation based on the properties of the JIA subtypes. Timely diagnosis and intervention are essential for maximizing quality of life, healthy growth and development, and prevention of long-term disability. This review aims to provide a clinically practical framework for the core elements important in recognition, monitoring, and management of JIA. **Methods**: We performed a narrative review of the current literature, complemented by real-world clinical experience from academic rheumatology practice. The review synthesizes evidence-based knowledge with practical insights to develop an approach that can be applied in daily clinical decision-making. **Results**: We propose a structured, stepwise method for evaluating suspected JIA, emphasizing the integration of pattern recognition with differential diagnosis. Our framework emphasizes two principal parameters: (1) the distribution of joint involvement (peripheral vs. axial) and (2) the presence of extra-articular manifestations, including uveitis, cutaneous findings, and gastrointestinal symptoms. This format aids in distinguishing major JIA subtypes and highlights their distinctive features. In addition, we review overarching principles for monitoring, assessing risk for uveitis, and treatment, and the importance of multidisciplinary care. **Conclusions**: This structured approach is intended to support clinicians in the accurate recognition of JIA and its subtypes, facilitate early diagnosis, and provide insights on management strategies that improve patient outcomes.

## 1. Introduction

Pediatric rheumatology is a derivative of Immunology that focuses on excessive inflammation caused by functional aberrations of leukocytes leading to an autoimmune, autoinflammatory, and immune-dysregulatory state of health. The diseases managed in this field include those affecting the musculoskeletal system, such as arthritis or myositis, as well as conditions affecting multiple organs (multisystem or systemic diseases), such as lupus, vasculitis, and scleroderma. Rheumatology is often engaged in multispecialty care in collaboration with organ-specific subspecialties. This allows the optimization of anti-inflammatory treatment of shared patients, including those with psoriasis, inflammatory bowel disease (IBD), or uveitis.

Juvenile Idiopathic Arthritis (JIA) is the most common pediatric autoimmune disease, accounting for approximately 40% of patients assessed by pediatric rheumatology [1,2]. JIA affects about 1 in 1000 children under the age of 16 years [3,4]. Although the incidence and prevalence of JIA is comparable to that of other rare pediatric diseases, early diagnosis and appropriate treatment of JIA remain challenging due, in part, to limited a pediatric rheumatology workforce globally [5].

This manuscript aims to convey the fundamental principles of JIA to primary care providers through a structured and systematic framework.

## 2. The Central Importance of Arthritis in Rheumatology

**Figure 1:**
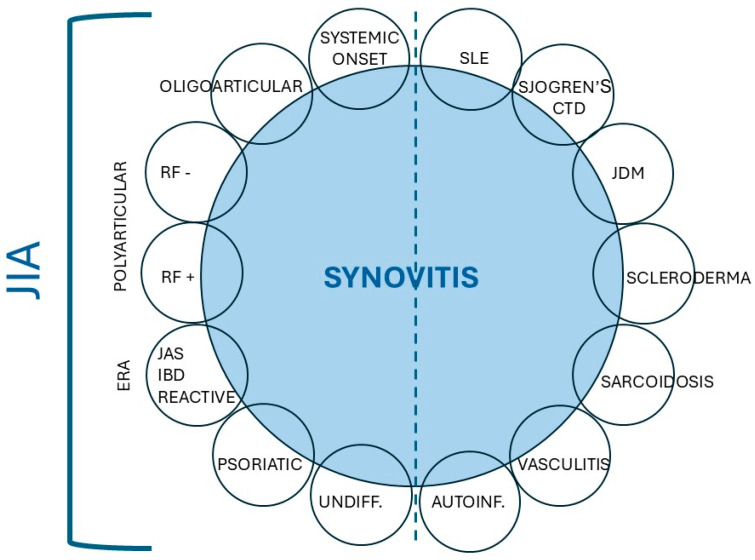
Arthritis, chronic inflammation of the synovium, can manifest as JIA or a part of systemic autoimmune disease; the two groups are separated by the dotted line. Abbreviations: SLE, systemic lupus erythematosus; CTD, connective tissue disease; JDM, juvenile dermatomyositis; Autoinf, autoinflammatory diseases; Undiff, undifferentiated arthritis; JAS, juvenile ankylosing spondylitis; IBD, inflammatory bowel disease; RF+, Rheumatoid Factor positive; RF−, Rheumatoid Factor negative.

In pediatrics, arthritis is often encountered as a common manifestation of various tissue pathologies caused by rheumatological conditions. As shown in Figure 1, it may present as a disease entity—JIA—that comprises several clinical subtypes. Arthritis can also be a feature of systemic autoimmune and inflammatory diseases. However, there is no single diagnostic test for any of the rheumatological diseases, including JIA; proper diagnosis is almost always based on pattern recognition. This diagnostic approach is abstractly analogous to solving a Rubik’s cube: identifying arthritis and its associated features serves as a diagnostic cornerstone to reach definitive conclusions.

## 3. Arthritis, Chronic Inflammation of Synovium

In general, arthritis refers to inflammation of the synovium, a lace-like, thin membrane located in the inner lining of the fibrous sheath that encapsulates diarthrotic joints, tendons, and bursae. According to the 1997 Durban classification criteria, JIA consists of seven distinct subtypes based on clinical properties [6], i.e., systemic-onset JIA (sJIA), oligoarticular JIA (also known as pauciarticular JIA), rheumatoid factor-negative (RF−) polyarticular JIA, rheumatoid factor-positive (RF+) polyarticular JIA, enthesitis-related arthritis (ERA), psoriatic arthritis, and undifferentiated arthritis. Although it is beyond the scope of this manuscript, the readers are encouraged to review the historical aspects of JIA classification, which has continued to evolve toward a global consensus among the pediatric rheumatology community since the 1970s. It is expected that the upcoming new classification will center on immunopathogenic and genetic properties of pediatric arthritis [7].

Regardless of the taxonomy, there is a common theme among different subtypes of JIA regarding the emergence of histopathological findings. The synovium is a unique tissue composed of two layers: the intimal and subintimal layers. The intimal layer is one to two cells thick and consists of macrophage-like, phagocytic type A cells and fibroblast-like, secretory type B cells. The subintimal layer is vascularized and contains many high endothelial venules [8]. Under normal conditions, joints are immuno-privileged sites; however, a disruption in tissue homeostasis can trigger inflammatory cascades.

In arthritis, the activation of endothelial cells leads to the recruitment of circulating blood leukocytes, resulting in chronic inflammation, hyperplasia, and thickening of the synovium. This process typically develops gradually over weeks, or even months, accompanied by the accumulation of a serous inflammatory milieu within the joint cavity. The inflammatory synovial fluid contains primarily neutrophils (<50,000/mm^3^), proteolytic enzymes, and cytokines. As a result, patients experience stiffness and dull, persistent pain in the affected joint(s), which is most noticeable in the mornings (i.e., morning stiffness). Additionally, tenderness upon palpation and pain with motion of the arthritic joint or tendon insertion sites may be present.

To minimize discomfort, children often avoid joint movement, which can lead to tendon tightness, joint contracture, and muscle atrophy. If untreated, patients with JIA may develop irreversible joint damage with bone and cartilage erosions, which can eventually lead to complete joint fusion over the years. While mortality associated with JIA is low, if the child does not receive adequate treatment, the morbidity and reduced quality of life due to long-term disability can be devastating [9,10].

## 4. JIA Is a Diagnosis of Exclusion—The Diamond Approach

There is no single test for JIA, and the conclusions must stem from diagnosis of exclusion. Rheumatologists are trained on pattern recognition using history and physical examination along with skillful laboratory investigations and, in some cases, imaging studies.

**Figure 2:**
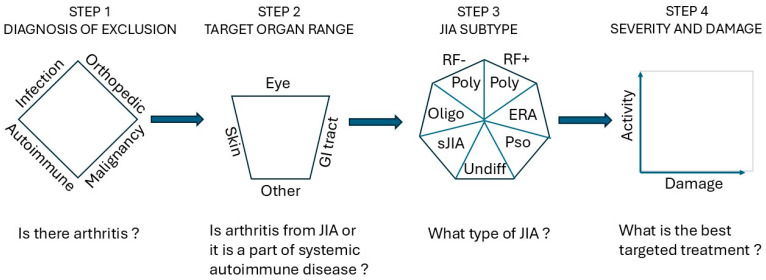
Clinical assessment of and conclusions on JIA require a stepwise approach built upon history and physical examination in a time continuum in conjunction with basic laboratory results and, where possible, images. The purpose of each step is given on the top line, and the decisions that must be made to move the next step are shown on the bottom line. ERA, Enthesitis-Related Arthritis; Pso, psoriatic arthritis.

As shown in Figure 2, the provider may apply the “diamond rule” to rule out infectious, malignant, or orthopedic conditions before considering arthritis when a child presents with joint complaints. The infectious disease (ID) work-up is most crucial to rule out septic arthritis when there is precipitous onset of severe joint pain, particularly in a febrile child with increased C-reactive protein (CRP). This is a medical emergency requiring care coordination with orthopedics and antibiotics. The ID work-up often includes serologies for streptococcus, Epstein–Barr virus (EBV), Lyme disease, and, sometimes, parvovirus for more gradual onset of arthropathy, over days or weeks. Orthopedic concerns include, but are not limited to, trauma and soft tissue injuries that often present with an acute-onset pain that may correlate with findings on imaging studies. In addition, hypermobility and alignment concerns (pes planus, pronated feet, etc.) commonly presents with intermittent transient joint complaints upon overuse that resolve in hours to days with rest. The possibility of malignancy manifesting with joint, back, or limb pain is most concerning and needs particular attention to signs such as nighttime awakenings or subtle abnormalities in the complete blood count (CBC). In these cases, early communication with hematology/oncology specialists is essential. It is necessary to continue close follow-up and monitor patients’ laboratory results, including CBC, lactate dehydrogenase (LDH), and uric acid levels. Patients may need abdominal ultrasound or a bone scan and, if in doubt, bone marrow examination [11].

## 5. JIA Versus Systemic Autoimmune Disease

For JIA, the diagnostic work-up always includes tests to evaluate the overall state of health, such as a CBC with differentials, complete metabolic panel (CMP), erythrocyte sedimentation rate (ESR), CRP, and urinalysis (UA). Additional laboratory tests, such as tests for iron and ferritin levels, vitamin D levels, thyroid function, and quantitative immunoglobulin levels, can provide further insights into the child’s overall condition. Specific laboratory tests, including antinuclear antibody (ANA) with reflex to a panel of autoantibodies for lupus, RF, cyclic citrullinated peptide (CCP) antibody, and HLA B27 (Human Leukocyte Antigen B27) can also be part of the initial work-up based on history and exam findings [12,13,14]. For selected patients, additional tests may include tests for angiotensin-converting enzyme (ACE), antineutrophil cytoplasmic antibodies (ANCA), or anti-Scl70 antibody and muscle enzymes when sarcoidosis, vasculitis, scleroderma, or myositis is suspected. Imaging studies, such as X-rays or MRI, may be required in specific cases to assess disease activity and damage, particularly for arthritis affecting the temporomandibular joints (TMJs), neck, wrists, hips, or sacroiliac joints.

## 6. Subtypes of JIA

Once there is confidence in the diagnosis of JIA, the next step is to determine the JIA subtype. The following parameters are important in decision-making:

### 6.1. Patient’s Age and Gender

Different types of arthritis tend to present at specific stages of life. Gender, as well as the number and distribution of affected joints, are also important factors. For instance, oligoarticular JIA is more common in girls, with a peak onset among toddlers, whereas enthesitis-related arthritis (ERA) more commonly affects boys around or after ages of puberty.

### 6.2. Number and Anatomical Distribution of Affected Joints

Oligoarticular JIA involves fewer than five joints and typically affects large joints with a peripheral distribution.

Polyarticular JIA involves five or more joints and may affect both small and large joints with bilateral symmetric joint distribution.

ERA, by contrast, often involves axial joint distribution centered on the sagittal plane, including the spine and large joints (e.g., hips, knees) near the spine. There is no strict limit on the number of joints affected, and patients may present with enthesitis with or without synovitis.

Systemic-onset JIA and psoriatic arthritis can display varying patterns and involve varying numbers of joints.

Undifferentiated arthritis is a type of arthritis that does not fit any of the above patterns.

These conditions may involve either peripheral or axial joints.

### 6.3. Type of Extra-Articular Target Organs Involved

The involvement of extra-articular organs such as the eyes, gut, and skin helps determining JIA subtypes. Inflammatory changes in these organs may emerge at different points in time in pediatric patients.

### 6.4. Laboratory Results and Family History

Laboratory Tests: Results of a few available tests, such as ANA, RF, CCP, and HLA-B27, can be helpful, but they are not definitive without clinical correlation. Some examples are given below:

ANA (antinuclear antibody) is positive in 15–17% of healthy children and does not indicate an increased risk of autoimmune disease in isolation [12].

RF and CCP (cyclic citrullinated peptide) antibodies are almost never positive in healthy children. Positive results require careful clinical investigation for arthritis or systemic autoimmune diseases [13,14].

HLA-B27 positivity may or may not indicate a risk for ERA; 8% of Caucasians are HLA-B27-positive, but very few develop arthritis [15].

Family History: Family history is often negative in sJIA, oligoarticular JIA, and RF-negative polyarticular JIA. However, it can provide strong supportive evidence for diagnosing psoriatic arthritis or ERA (e.g., ankylosing spondylitis or inflammatory bowel disease-associated arthritis). RF-positive JIA patients may have adult family members suffering from rheumatoid arthritis.

## 7. A Brief Overview of JIA Subtypes for Overall Clinical Characteristics

**Figure 3:**
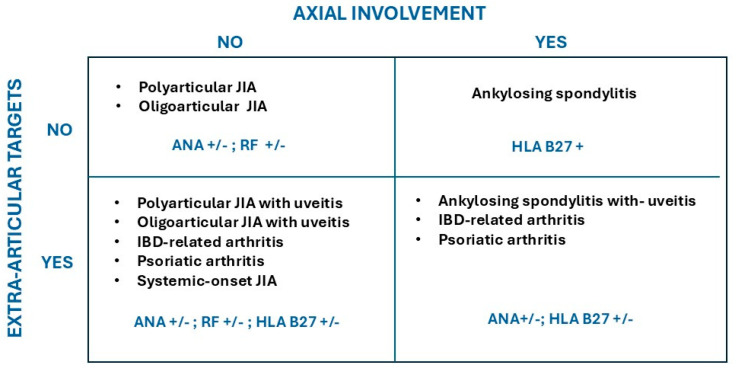
Simplified overview of the subtypes of arthritis based on two properties: distribution of affected joints, and presence or absence of extra-articular target organ(s). HLA B27 (Human Leukocyte Antigen B27) is most associated with axial involvement.

Figure 3 depicts a simplified outline of the different types of JIA. The two dimensions include the symmetry of the distribution of the affected joints within the horizontal or axial anatomical planes, and whether there is co-presence of an extra-articular tissue.

The following are the clinical highlights of different types of JIA as summarized in Table 1:

**Systemic-onset JIA (sJIA)** is also referred to as Still’s disease, named after the British physician who first described the condition in children in 1897 [16]. Later, several diagnostic criteria were developed for adult-onset Still’s disease, with Yamaguchi’s criteria being the most widely recognized [17]. It can occur at any age, even as early as infancy.

In practice, in the absence of an infectious etiology, four hallmark findings strongly suggest a diagnosis of sJIA: fever, rash, arthritis, and specific CBC findings [18]. The fever associated with sJIA is termed quotidian fever, characterized by spikes as high as 104 °F or more once or twice daily, typically occurring in the late evening or early morning. These fever spikes are transient and resolve spontaneously, even without treatment. The rash associated with sJIA typically occurs during fever spikes and is described as salmon-colored, non-palpable, and evanescent (i.e., it comes and goes). Patients often exhibit dermographism that is not associated with histamine release and is called the Koebner phenomenon.

Arthritis in sJIA can involve any number of joints, from as few as one to more than 30, including the cervical spine, TMJ, and even cricothyroid joints. The typical CBC findings necessary for a confident diagnosis include leukocytosis and thrombocytosis. Patients often present with anemia and elevated inflammatory markers, such as ESR and CRP, consistent with anemia of chronic disease rather than anemia caused by nutritional deficiencies, consumption, or malignancy.

sJIA may also present with additional systemic manifestations, including lymphadenopathy, hepatosplenomegaly, and serositis affecting the heart and/or lungs, which generally correlate with disease severity. Pericarditis in sJIA can be severe enough to cause cardiac tamponade, requiring pericardial drainage unless the patient receives prompt anti-inflammatory treatment.

sJIA can become life-threatening with the onset of macrophage activation syndrome (MAS). For an in-depth discussion of MAS, please refer to a recent review [19]. Systemic-onset JIA is classified as a non-monogenic autoinflammatory disease, with varying severities of IL-1 and IL-18 pathway activation. The advent of biologic response modifiers targeting IL-1 and IL-6, as well as Janus kinase (JAK) inhibitors, has significantly improved disease control and long-term outcomes.

**Oligoarticular (i.e., Pauciarticular) JIA** is considered the simplest form of JIA, though it has unique clinical aspects that require consideration [20]. As described above, children with oligoarticular JIA present with arthritis affecting up to four joints, most commonly the knees, ankles, wrists, and elbows, which may be involved unilaterally or bilaterally. These are typically toddlers who are otherwise healthy, with no history of fever, rash, or chronic gastrointestinal concerns.

These children often present with stiffness, sometimes accompanied by a mild limp in the mornings, which becomes less apparent later in the day. There is no associated fever or rash. The child overall appears healthy, keeping up with active daily life. The onset of symptoms can be quite insidious, and it may first be noticed after chronic changes have developed, such as bony enlargement of the joint, flexion contractures, muscle atrophy, or leg length discrepancies. Oligoarticular JIA can progress to affect several joints over time, described as “extended oligoarticular (or extended pauciarticular) JIA”.

Basic laboratory tests, such as CBC and CMP, are typically normal, and ESR and CRP levels may or may not be elevated. It is not uncommon for these children to undergo joint aspiration, particularly if they first present to the emergency department or Orthopedics. The synovial fluid typically shows white blood cell counts ranging from 5000 to less than 50,000/mm^3^, with negative results for Gram-stain, routine cultures, and Lyme PCR.

Testing for ANA is important, as ANA positivity is a prognostic marker for an increased risk of uveitis. About 10 to 20% of patients with JIA are at risk for developing uveitis [21], often with a chronic course, as detailed below. It is important to note that the clinical progression of arthritis and uveitis, as well as their responses to treatment, may not always correlate to one another; for treatment decisions, although both are treated concurrently, management of uveitis generally takes priority over that of arthritis.

**Polyarticular JIA** accounts for approximately 40% of all cases of JIA, with a bimodal distribution of age at onset, peaking during toddler years and again in preadolescence or early teenage years [22]. The onset of polyarticular JIA can range in intensity from mild to severe, with some children becoming bedridden. Symptoms may progress rapidly within a few weeks or develop gradually over months.

These children often present with dull, constant arthralgia and prolonged morning stiffness. Other vague symptoms may include fatigue, poor appetite, weight loss, reluctance to participate in physical education classes and activities, depressive mood, and missed school days. Difficulty with both fine and gross motor skills is common, impacting self-care and ambulation. Hip joint involvement further escalates concerns regarding long-term disability and potential risk of requiring a wheelchair.

When a preteen or older child presents with polyarticular arthritis, it is crucial to differentiate JIA from other systemic autoimmune diseases, such as lupus (see Figure 1). Serologically, approximately 10% of children with polyarticular JIA test positive for RF, which is an autoantibody (most commonly of the IgM class) that targets the Fc portion of the IgG antibodies. RF positivity signifies early-onset adult-type rheumatoid arthritis (RA). It is also a prognostic factor for a course that is often severe, persistent, and erosive arthritis.

It is important to note that RF can also be positive in other conditions, such as lupus, Sjögren’s syndrome, sarcoidosis, and certain cancers or chronic infections (e.g., tuberculosis, bacterial endocarditis, and viral hepatitis). These conditions are typically distinguishable by their clinical presentations and/or additional laboratory markers. Anti-cyclic citrullinated peptide (CCP) antibodies are more specific to rheumatoid arthritis and may be positive in children with RF-positive polyarticular JIA.

While both RF and CCP are markers of severe JIA, pediatric patients rarely develop systemic complications such as rheumatoid nodules, or rheumatoid vasculitis that can be associated with severe forms of RA among adults [23].

**Enthesitis-Related Arthritis (ERA)** is a subtype of JIA distinguished by its demographics and clinical characteristics. ERA more commonly affects boys older than six years and typically manifests with arthritis and/or enthesitis, often in an axial distribution. According to the published criteria for ERA [24], the hallmark site of arthritis is the sacroiliac (SI) joints, particularly in HLA-B27-positive patients. However, in children, the development of MRI-confirmed SI arthritis—necessary for a diagnosis of juvenile ankylosing spondylitis (JAS)—may take months to years. During this time, these patients’ clinical findings may mimic oligoarticular JIA.

Enthesitis refers to the inflammation of the tendon where it inserts into the bone. Commonly affected sites include the Achilles tendon insertion site on the calcaneus and the tendons in the plantar fascia. Other commonly involved areas are tendons around the knees, hips, pelvic brim, elbows, shoulder joints, and costosternal junctions.

The disease activity in ERA is influenced by the gut microbiome and gut-associated lymphoid tissues (GALT), as evidenced by its association with IBD and reactive arthritis. Unlike oligoarticular, RF-negative polyarticular, or sJIA, ERA often has a familial pattern. Patients may have a family history of chronic back pain, ankylosing spondylitis, or IBD.

Distinct MRI findings of SI joint inflammation and/or the presence of acute anterior uveitis are uniquely associated with ERA. However, their presence is not strictly required for diagnosis.

**Juvenile Psoriatic Arthritis (JPsA)** is the most heterogeneous type of arthritis within JIA, exhibiting variability in age of onset, distribution of affected joints, extra-articular involvement, and family history. It accounts for approximately 10% of JIA cases and has a bimodal age of onset: toddlers and older children in late elementary school years or adolescence.

Toddlers with JPsA are usually girls and often present with arthritis resembling other forms of JIA (oligoarticular or polyarticular). However, hallmark features may distinguish JPsA, including the following:

The presence of dactylitis (sausage-like swelling of an entire digit), involvement of distal interphalangeal (DIP) joints, and asymmetric joint distribution.

Older children with psoriatic arthritis may exhibit features more similar to those of enthesitis-related arthritis (ERA), such as axial joint involvement, sacroiliac (SI) arthritis, and enthesitis.

While psoriatic arthritis can be associated with skin and nail changes (e.g., psoriasis, nail pitting), these are not universally present. The disease course of JPsA can range from self-limiting and transient to rapidly progressive and mutilating.

Children with JPsA often have a positive family history of psoriasis. Blood tests may show positive ANA serology; however, RF and HLA-B27 are typically negative [25].

**Undifferentiated arthritis** includes patients who do not fulfill all the criteria for one or the other six types of JIA or fit more than one [26].

## 8. Extraarticular Target Organs Involved in JIA

Certain subtypes of JIA have hallmark associations with inflammation in extra-articular target organs, including the eyes, intestinal tract, and skin. The severity of inflammation at these remote sites can vary significantly. Additionally, the timing of inflammation in articular and extra-articular tissues is not always synchronized. In other words, the onset of arthritis may precede, coincide with, or follow the emergence of inflammation in other target organs.

**Eyes:** The uveal tract, the middle layer of the eye located between the sclera and retina, can develop inflammation in JIA [27]. The uvea comprises the iris, ciliary body, and choroid. In JIA, inflammation typically manifests as anterior uveitis, which occurs in the anterior chamber of the eye, between the cornea and iris.

Different subtypes of JIA are associated with distinct patterns of uveitis:

Oligoarticular, RF-negative polyarticular, and psoriatic JIA often cause chronic anterior uveitis, which has an insidious onset and gradual progression.

Enthesitis-related arthritis (ERA), by contrast, is more commonly associated with acute anterior uveitis (AAU), characterized by acute onset, redness, and painful eyes.

Uveitis is relatively common, occurring in up to one in every five children with JIA, and it may affect one or both eyes. Children < 7 years of age and with positive ANA have the highest risk for uveitis [28]. The American Academy of Ophthalmology recommends regular eye exams of JIA patients by pediatric ophthalmologist up to four times per year [29]. This is essential, as chronic progression of uveitis, if undetected, can lead to permanent damage, including synechia of the iris, band keratopathy of the cornea, cataracts, and glaucoma that can lead to blindness over time.

If uveitis is diagnosed, the patient is followed very closely both by ophthalmology and rheumatology. Treatment typically begins with steroid eye drops under ophthalmology guidance. Based on the severity and persistence of the uveitis, systemic treatment is promptly escalated, even in cases of mild arthritis.

**Gastrointestinal (GI) Tract:** Inflammation within the GI tract is often associated with chronic arthritis, affecting individuals of all ages, including children [30]. This is most often observed in patients with IBD, such as Crohn’s disease and ulcerative colitis. IBD-related JIA typically manifests as enthesitis-related arthritis (ERA) with axial joint involvement. However, as noted earlier, in young children or during the early course of the disease, the arthritis may present with a peripheral distribution resembling oligoarticular or polyarticular JIA.

The severity and activity of JIA often correlate closely with the degree of inflammatory changes in the GI tract. Monitoring typically includes assessing stool calprotectin as an indicator of intestinal inflammation, alongside routine laboratory tests such as CBC, chemistries, ESR, and CRP.

Treatment is generally tailored to control IBD through a multidisciplinary approach, ensuring that both the gastrointestinal and articular components of the disease are managed effectively.

**Figure 4:**
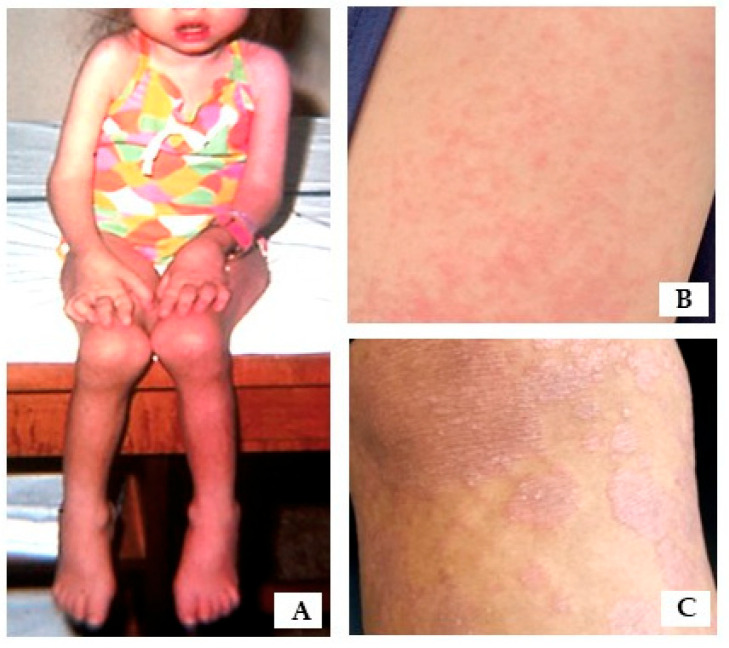
(**A**). A toddler with sJIA affecting multiple joints, note symmetric involvement of small and large joints throughout with signs of disease damage, including petite size, micrognathia, joint contractures, Boutonnière deformity of fingers, wrist drop, muscle atrophy and flat affect without acute distress or pain. This is a historical picture to illustrate the potential consequences of JIA prior to the era of bDMARDs (photo provided by DKM). (**B**). Salmon colored, evanescent, non-palpable sJIA rash. (**C**). Psoriatic rash on a knee.

**Skin:** The rash (Figure 4) associated with sJIA typically occurs during fever spikes and is described as salmon-colored, non-palpable, and evanescent (i.e., it comes and goes). Patients often exhibit dermographism. Psoriasis-related skin findings, which can vary in severity and include changes such as nail pitting, discoloration, or thickening, as well as the characteristic psoriatic rash, which typically presents as inflamed, erythematous, raised, and scaly plaques [31]. Overall, about one-third of patients with psoriatic disease experience both joint and skin manifestations, another one-third present with only psoriatic skin rash, and the remaining one-third have psoriatic arthritis without visible skin involvement. In cases where psoriatic arthritis occurs without skin findings, a family history of psoriasis can be particularly helpful in making the diagnosis. Care for these patients is usually coordinated with dermatology. In the presence of arthritis, treatment decisions are primarily managed by rheumatology. Treatment regimens are typically tailored to control arthritis, unless skin manifestations are severe and inadequately managed with topical therapies alone.

## 9. Longitudinal Assessment of Disease Activity and Damage

The methodology for formulating a disease activity score is essential for accurately assessing treatment response in everyday clinical practice. For patients with an established diagnosis of juvenile arthritis, longitudinal assessment of disease severity correlates most objectively with the number of affected joints and the extent of swelling in each arthritic joint. Although these assessments are subject to interobserver variability, careful and consistent documentation is critical.

Some clinicians prefer shorthand notation using the acronym “SPLT”, which stands for Swelling, Pain, Limitation, and Tenderness.

Swelling is the palpation of intraarticular fullness as bloated fluid or synovial thickening.

Pain refers to discomfort during active or passive range of motion. Tenderness describes discomfort upon palpation of the joint.

Limitation is the restriction of natural joint range upon active (with provider’s assistance) and passive (without provider’s assistance) movement.

Swelling, pain, and tenderness are graded from 0 (none) to 3 (worst).

Limitation is graded on a scale from 0 to 4, reflecting a 25% incremental limitation in range of motion (i.e., <25%, 25%, 50%, 75%, or 100% loss in joint range).

To simplify documentation, some providers use graphical tools, such as a homunculus diagram available online, to record joint involvement.

In routine practice, rheumatologists also assess the following parameters:

Pain, measured as severity on a 0 to 10 scale (10 being the worst), often using a Visual Analog Scale (VAS).

Stiffness, measured as duration in minutes.

Functional limitations, typically evaluated using the Childhood Health Assessment Questionnaire (CHAQ) [32].

Quality of life, assessed with tools like the Pediatric Quality of Life Inventory (PedsQL).

Trends in inflammatory markers, such as CRP and ESR, are helpful for monitoring disease activity and response to treatment, especially if baseline levels were elevated. Children with mild to moderate severity of JIA, however, may have normal CRP and/or ESR at initial presentation.

In addition, a patient/parent or physician global assessment of overall disease impact is measured using VAS (0 to 10, with 10 being the worst).

Scoring systems combining these tools have been validated in research and are commonly used as outcome measures in clinical trials. Examples include the American College of Rheumatology (ACR) JIA response criteria [33], and Juvenile Arthritis Disease Activity Score (JADAS) [34].

These scoring systems help standardize assessments and guide treatment decisions [35,36].

## 10. Current Treatment Guidelines

There is no cure for arthritis; the primary goal is to halt synovial inflammation and prevent joint damage in a growing child (Figure 4). Most therapeutic agents target innate immunity and proinflammatory cytokines due to their fundamental role in immunopathogenesis of arthritis across different types. There are some general rules worth mentioning: First, accomplishing control over an autoimmune disease is a stepwise process, i.e., remission on treatment followed by remission on minimal treatment, and finally, if possible, remission off treatment. Second, this objective requires continuous titration and tailoring treatment based on disease severity and damage, co-morbidities, compliance, availability of pharmaceutical agents, and response to treatment. Third, during the course, pro re nata (PRN) use of systemic steroids is strongly discouraged both prior to rheumatology evaluation and also as part of a chronic treatment program. Lastly, while treatment protocols for JIA have been well-documented [37,38,39], they remain a moving target due to the rapid emergence of new therapeutics [40]. For some cases, in-patient management may be necessary, particularly for those with sJIA who may need intra-venous (IV) infusions and/or close monitoring for serositis or MAS. The following summarizes common ambulatory methods for the management of JIA to control synovial inflammation.

First-Line Treatment:

Non-steroidal anti-inflammatory drugs (NSAIDs) are typically the first-line treatment, administered daily during the initial weeks while evaluating the type and severity of arthritis. NSAIDs inhibit cyclooxygenase enzymes (COX) and down regulate prostaglandin synthesis. COX1 inhibitors, such as ibuprofen (10 mg/kg/dose) TID or naproxen (5–10 mg/dose BID), are commonly used for a trial period of up to 8–10 weeks. If there are concerns about dyspepsia or platelet dysfunction, COX2 inhibitors, such as Celecoxib, are preferred. NSAIDs alone or in combination with intra-articular injections of long-acting steroids, such as Triamcinolone acetonide (i.e., Kenalog), can be effective in children with oligoarticular JIA. The dose of triamcinolone ranges from 3 to 5 mg (for the PIP joint) to 20 to 40 mg (for the knee) based on the estimated joint surface area. It is often mixed with 1% lidocaine 1:1 by volume prior to injection. Intra-articular injection can be repeated every 3 to 4 months but is considered a temporary measure.

Advanced Therapies:

In patients with polyarticular JIA, persistent oligoarticular JIA, ERA, or psoriatic arthritis with significant joint or skin involvement, or with the presence of uveitis, the treatment should be escalated to Disease-Modifying Antirheumatic Drugs (DMARDs) promptly under the guidance of a rheumatologist. There are three main classes of DMARDs:

Conventional Synthetic DMARDs (csDMARDs): Methotrexate has been most used in the last four decades. It is a chemotherapy given weekly via an oral or subcutaneous route, usually up to 25 mg/dose or 30 mg/m^2^/dose. Methotrexate is also used by several specialties, including oncology (at high doses) as well as dermatology, ophthalmology, and gastroenterology (at similar doses). The mechanism of action is multifaceted, including interfering with de nova purine and thymidylate synthesis, as well as intracellular adenosine signaling. This allows modulation of leukocytes as well as synovial fibroblasts. Methotrexate may, however, be insufficient to prevent chronic changes in target (joint, eye, skin, or gut) tissues and can lead to bone marrow suppression and increased liver enzymes, requiring monitoring of labs every 2 to 3 months. Patients are usually prescribed folic acid supplement (1 mg daily except on methotrexate days) to minimize CBC changes. It is important to counsel teens on iatrogenic risks of methotrexate, which can cause liver damage upon drinking and fetal defects or fetal loss upon pregnancy. Other DMARDs include leflunomide, azathioprine, sulfasalazine, and hydroxychloroquine, with more limited use in treatment of JIA.

Biologic DMARDs (bDMARDs): With the advent of biologics in late 1990s, it has been possible to reach remission in moderate to severe arthritis [41]. This class of therapeutics are monoclonal antibodies, or recombinant proteins with high specificity and efficacy to bind and neutralize soluble proinflammatory cytokines or block their receptor binding to inhibit downstream inflammation. bDMARDs revolutionized the field, as they enabled delivery of an inflammatory-pathway-specific treatment. Those designed against TNF-alpha were the first prototypes, and are the most widely used to date. They can be used alone or in combination with methotrexate for treatment of different types of JIA and uveitis. Those targeting IL-1 and IL-6 are the first-line biologics for treatment of sJIA. Anti-IFN gamma is available, and anti-18 formulations are in the pipeline, for treatment of MAS. Some of the recent biologics directed against IL-23 are used in RF-positive arthritis, ERA, psoriasis, and IBD. B lymphocyte depletion by anti-CD19 is used more commonly in adults with RA.

Targeted Synthetic DMARDs (tsDMARDs): Within the last two decades, Janus kinase enzyme inhibitors (JAKis) have been marketed as a new class of therapeutics for treatment of autoimmune diseases, including arthritis. Unlike protein-based biologics that require a parenteral route, JAKis are synthetic molecules given as daily oral pills. The mechanism of action involves blocking intracellular signaling of type I and type II cytokine receptors upon binding of a range of proinflammatory cytokines, including IL-2, IL-6, IL-12, IL-23, interferon-γ, and granulocyte-macrophage colony-stimulating factor (GM-CSF). The specificity, and thus the biological effect, is determined by targeted inhibition of different JANUS kinase enzymes.

The most significant adverse effect of DMARDs is infection. Some biologics and JAKis require monitoring labs on CBC, liver enzymes, and lipid profile. As a universal precaution, screening for tuberculosis and hepatitis is required prior to initiating these medications and should be checked yearly.

Comprehensive Care:

Effective management of JIA requires a multidisciplinary approach in collaboration with the primary care team. Alongside rheumatology and ophthalmology, the child may benefit from physical and occupational therapy, nutrition support, counseling/child life specialists, social work, and, when needed, consultations with gastrointestinal or dermatology services.

## 11. Role of Primary Care Provider for a Child with JIA

Juvenile Idiopathic Arthritis (JIA) is a chronic condition that requires a multispecialty medical team to achieve the best long-term outcomes. The primary care provider serves as part of the collaborating team among specialists, including rheumatology, ophthalmology, physical and occupational therapy, and counseling services.

**Monitoring and Laboratory Work:** Regular monitoring of growth and development as well as vigilance for medication-related adverse effects is crucial. Laboratory evaluations are often needed every 2 to 3 months and typically include CBC, CMP, ESR, CRP, and urinalysis.

Specialized tests, such as tests for blood levels of therapeutics and screening for autoantibodies against therapeutics, may be required under the guidance of a rheumatologist.

**Preventive measures against infection and early intervention:** Children receiving immunomodulators/DMARDs for JIA are immunosuppressed and subject to susceptibility and/or escalated severity of infection. Awareness and, if there is any doubt, prompt and proper intervention with any signs and symptoms of infection are of the utmost importance in these children’s care. Families should seek urgent care for any fever and inform the medical team of their child’s progress. During an infection, it is often recommended to reduce or hold the dose of immunomodulators to allow recovery. In addition, some infections, such as Streptococcal pharyngitis, mycoplasma, shigella, salmonella, and EBV, can trigger a JIA disease flare, and post-infection follow-up may be needed.

**Family and Psychosocial Support:** Providing support to families is essential to address concerns related to coping, mood, and peer relationships, which significantly impact quality of life. Special attention should be given to minimizing exposure to illness and ensuring prompt evaluation and treatment of infections, especially in the presence of fever.

**Immunizations:** Routine immunizations and annual flu vaccine are generally encouraged among children with JIA. It is best to administer vaccines when the child is clinically stable and the underlying disease is well-controlled. Live-virus vaccines should be avoided while the patient is receiving DMARDs. The timing of vaccines should sometimes be adjusted relative to immunosuppressive dosing; as an example, for a child on a biologic with infusions/injections every 2–4 weeks, the vaccine may be given midway between doses to maximize the immune response. For patient-centered care, coordinating decisions with the treating rheumatologist will be optimal.

**Communication between school and family on child’s wellbeing and community effort towards education and advocacy:** Children with JIA are often bright and high achievers in school. By law, they are entitled to an Individualized Education Plan (IEP), which provides accommodations such as modified curricula and additional resources if needed. IEP should also include communication with the family to warn of any infectious outbreaks in the classroom and, when indicated, allow school absences to avoid exposure. Advocacy support by primary care teams provides extra support for patient and parent education and community engagement, which are essential for holistic healing of not only the child, but also the family. Online resources are available that can assist primary care providers to gain further insights.

**Workforce and future directions:** Pediatric rheumatology, a subspecialty of the American Board of Pediatrics, requires three years of fellowship following general pediatrics training. In the United States, there are approximately 600 board-certified pediatric rheumatologists and 40 accredited fellowship programs; the global workforce is estimated to be <10,000 [5]. Despite workforce limitations, the field is advancing rapidly, with the adoption of next-generation therapeutics and expansion of multicenter translational research. Clinical outcomes have improved substantially over recent decades, accomplishing near elimination of wheelchair dependence among children with severe JIA [42]. Future protocols are expected to emphasize sustained remission and the pursuit of curative therapies.

It is important to note, however, that many bDMARD and tsDMARD agents currently available on the market lack U.S. Food and Drug Administration (FDA) approval for pediatric indications. Industry efforts to expand pediatric labeling remain limited, constrained by financial considerations and a small market size. This gap represents a central challenge in the field and compels pediatric rheumatologists, in partnership with families, to seek strategies that accelerate regulatory approval pathways and ensure timely access to effective therapies [43].

**Disclaimer** The views expressed in this article are those of the authors and do not necessarily reflect the official policy of the Department of Defense or the U.S. Government. The identification of specific products or scientific instrumentation is considered integral to this scientific endeavor and does not constitute endorsement or implied endorsement on the part of the authors, DOD, or any component agency.

## 12. Summary

1. Juvenile Idiopathic Arthritis (JIA) is the most common pediatric rheumatologic disease, driven by chronic synovial inflammation and capable of causing long-term disability.

2. Diagnosis is clinical and by exclusion; no single test confirms JIA. Laboratory markers such as ANA, rheumatoid factor, and HLA-B27 are not diagnostic but offer prognostic value for uveitis risk, arthritis severity, and sacroiliac involvement, respectively.

3. JIA comprises multiple subtypes defined by joint count and distribution, systemic features, and extra-articular organ involvement.

4. Systemic-onset JIA is distinguished by quotidian fever, rash, arthritis, and CBC abnormalities, and carries a risk of macrophage activation syndrome (MAS), a potentially life-threatening complication.

5. Uveitis is the most frequent extra-articular manifestation and a major cause of acquired blindness in Western countries. Often asymptomatic, it necessitates ophthalmologic screening every 3–12 months based on risk factors (age <7 years, ANA positivity).

6. Management requires rheumatology oversight. Treatment is often individualized and ranges from NSAIDs to immunomodulators; intra-articular steroid injection can benefit oligoarticular JIA, while systemic steroids are reserved for selected systemic-onset JIA and MAS. Regular clinical assessment and outcome tracking are essential.

7. Primary care providers are critical for early recognition, infection and drug toxicity monitoring, multidisciplinary coordination, school liaison, and holistic, family-centered support to optimize growth and functional outcomes. 

8. These children are often immunocompromised while receiving treatment, even if their white blood cell count is normal. Therefore, live virus vaccines should be avoided. It is also of utmost importance to promptly recognize and appropriately manage any signs of fever or infection throughout the course of therapy.

## Figures and Tables

**Table 1:** Clinical highlights of JIA subtypes.

JIA Subtype	History of Present Illness	Distinct Clinical Characteristics	Physical Exam	Labs	Red Flag	Treatment
**OLIGO** **ARTICULAR**	Chronic days in and out, dull pain, and morning stiffness; usually toddlers F > M.	Most common form of JIA (about 40%), with risk for uveitis, particularly if ANA is positive; about 30% grow out of it.	Total joint count < 5; usually affects knees, ankles, wrists, or elbows; eating, sleeping, and playing well.	Expect normal CBC, CMP, and UA; ESR and CRP can be normal or mildly increased; ANA can be positive in 40% cases.	Uveitis can lead to ocular damage; joint damage can lead to leg length discrepancy and tendon tightness	NSAIDs and/or intra-articular steroid (triamcinolone) injection.
**POLY** **ARTICULAR RF-NEGATIVE**	Chronic days in and out, dull pain, and prolonged morning stiffness; ages: young children to preteens/teens. F > M.	About 30–40% of JIA; Often with chronic course, poor quality of life and significant impact on daily activities; Risk for uveitis, particularly if ANA is positive; about 30% eventually grow out of it.	Total joint count ≥ 5 joints; usually symmetric with horizontal distribution.	Expect normal CBC, CMP, and UA; there might be anemia of chronic illness; ESR and CRP are usually increased; ANA can be positive in 40% of cases.	Patient needs close follow-up and careful treatment to avoid stunted growth, erosive joint changes and long-term disability.	Brief course of NSAIDs that is rapidly escalated to DMARDs and/or biologic response modifiers.
**POLY** **ARTICULAR RF-POSITIVE**	Chronic days in and out, dull pain, and prolonged morning stiffness; ages: usually teens. F > M	Childhood age onset of adult type Rheumatoid Arthritis. Almost always severe and cause significant impact on daily life.	Total joint count ≥ 5 joints; symmetric horizontal distribution.	RF is positive; expect normal CBC, CMP, and UA; there might be anemia of chronic illness; ESR and CRP are usually increased; ANA can be positive.	Patient needs aggressive treatment to avoid progressive and erosive arthritis into adulthood; high risk for stunted growth and long-term disability.	DMARDs and/or biologic response modifiers.
**SYTEMIC-ONSET**	Must have fever, rash, and arthritis; severe cases may have lymphadenopathy, hepatosplenomegaly, or pleural or pericardial effusion; any age.	Quotidian fever pattern, i.e., recurrent fever spike once or twice a day, afebrile in between; salmon-colored non-palpable evanescent rash usually found during fever.	Total joint count ≥1, often horizontal distribution.	Leukocytosis and thrombocytosis; may have anemia of chronic illness; often high ESR and CRP.	Risk for macrophage activation syndrome that can mimic sepsis hallmark findings, including pancytopenia, as well as increased blood LFTs, ferritin, LDH, and d-dimer.	Treatment often requires steroids and bDMARDs.
**ERA**	Patients often present with chronic joint and/or back pain. Reactive arthritis usually with acute onset following infection/gastroenteritis. Usually M > F of preteen age and older. FH is often positive for back pain or IBD.	Enthesitis and sacroiliitis are common. There might be GI concerns and weight loss, low-grade temperature, or fatigue if it is associated with IBD.	Total joint count may vary; typically associated with enthesitis and axial distribution of arthritis that may include sacroiliac joints, particularly if HLA B27 is positive.	There might be anemia and high inflammation markers (ESR, CRP), particularly if there is IBD. HLA B27 is often positive, but negative HLA B27 does not rule out ERA.	SI arthritis is usually progressive and can lead to disability. ERA can cause acute anterior uveitis with red, painful eyes. It can also cause chronic uveitis. GI involvement can be severe and may precede, co-present with, or proceed arthritis.	Treatment often requires bDMARDs.; reactive arthritis may be treated with NSAIDs or brief course of steroids.
**PSORIATIC**	Chronic days in and out, dull pain, and morning stiffness that may vary in severity over time. Age of onset: young children to preteens/teens, F ≥ M; FH often positive for psoriasis.	Course can be fluctuating in severity, usually responding well to treatment, or it can be of mutilating severity.	Total joint count may vary. It may have horizontal or axial distribution of arthritis that may also include sacroiliac joints.	ANA can be positive; RF or CCP are negative; HLA B27 is negative.	It can be associated with uveitis; skin/nail involvement can be severe and may precede, co-present with, or proceed arthritis.	Based on severity of arthritis and skin rash, treatment can range from NSAIDs to DMARDs.
**UNDIFFERENTIATED**	Joint pain and stiffness with varying pattern.	Varying clinical findings.	Total joint count may vary.	Varying labs.	Varying course and properties.	Varying treatment based on patient’s presentation.

RF, rheumatoid factor; ANA, anti-nuclear antibody (titer ANA usually <1:640, with low threshold to expand labs to rule out lupus if ANA is highly positive, or other systemic diseases); LFTs, liver function tests; NSAIDs, non-steroidal anti-inflammatory drugs; DMARDs, disease-modifying anti-rheumatic drugs; bDMARDs, biologic DMARDs.

## Data Availability

No new data were created or analyzed in this study. Data sharing is not applicable to this article.
